# Association between inferior posterior staphyloma on choroidal vessels running patterns in healthy eyes

**DOI:** 10.1186/s40942-025-00661-w

**Published:** 2025-03-27

**Authors:** Hiroto Terasaki, Ryoh Funatsu, Koki Okamura, Naohisa Mihara, Hideki Shiihara, Takehiro Yamashita, Shozo Sonoda, Taiji Sakamoto

**Affiliations:** Department of Ophthalmology, Kagoshima University Graduate School of Medical and Dental Sciences, Kagoshima, Japan

**Keywords:** Haller’s vessels running patterns, Choroid, Macular shape, Inferior posterior staphyloma

## Abstract

**Background:**

Effects of macular shape changes on the retina have been studied in pathologic myopia. However, whether there are individual differences in macular shape in non-pathologic myopia and the influence of macular shape on retinochoroidal disease in these eyes is not well known. A recently developed ultra-wide-field optical coherence tomography (UWF-OCT) has a wider imaging range and can be used to evaluate inferior posterior staphyloma (IPS). We aimed to investigate the effect of IPS on Haller vessel running patterns (HVRPs) in healthy eyes using UWF-OCT.

**Methods:**

This single-center retrospective study included healthy subjects. UWF-OCT images of normal subjects were stretched vertically to enhance the macula's shape and classified into IPS (n = 16) and non-IPS (n = 113) groups with or without propensity score matching (PSM) for age, sex, and ocular axis length. The HVRPs were subjectively classified into symmetry, superior dominant, and inferior dominant. Differences in the proportions of the patterns between the two groups were compared using Fisher's exact test.

**Results:**

In the non-IPS group, 65 (57.5%) individuals had a symmetric pattern of Haller's vessels, 32 (28.3%) had an upper-dominant pattern, and 16 (14.1%) had a lower-dominant pattern. In the IPS group, 14 eyes (87.5%) presented an upper dominant pattern, and 2 (12.5%) presented a symmetric pattern. There was a significant difference in vascular running patterns between the two groups (P < 0.001). After the PSM, a similar trend was confirmed.

**Conclusions:**

The eyes with IPS are likely to have superior dominant HVRPs compared to the non-IPS group in healthy eyes. Macular shape may play a role in HVRPs, which are involved in the pathogenesis of retinochoroidal diseases.

**Supplementary Information:**

The online version contains supplementary material available at 10.1186/s40942-025-00661-w.

## Background

The effects of macular shape changes on the retina have been studied in pathologic myopia, such as retinoschisis [1–4], macular hole retinal detachment [1–4], and serous retinal detachment in the macula associated with an inferior posterior staphyloma (IPS) or dome-shaped macula [5–8]. However, whether there are individual differences in macular shape in non-pathologic myopia and the influence of macular shape on retinochoroidal disease in these eyes remains largely unknown. Our group studied individual differences in macular shape in optical coherence tomography (OCT) images of healthy eyes of elementary and junior high school students using a method to enhance the macular shape by vertically stretching the OCT images [9]. Even in healthy eyes, we found individual differences in macular shapes, such as cone-shaped, flat-shaped, and dome-shaped [9]. Using this technique, our group reported that macular shape affected the findings (hole diameter and basal hole diameter) of idiopathic macular holes [10].

**Fig. 1:**
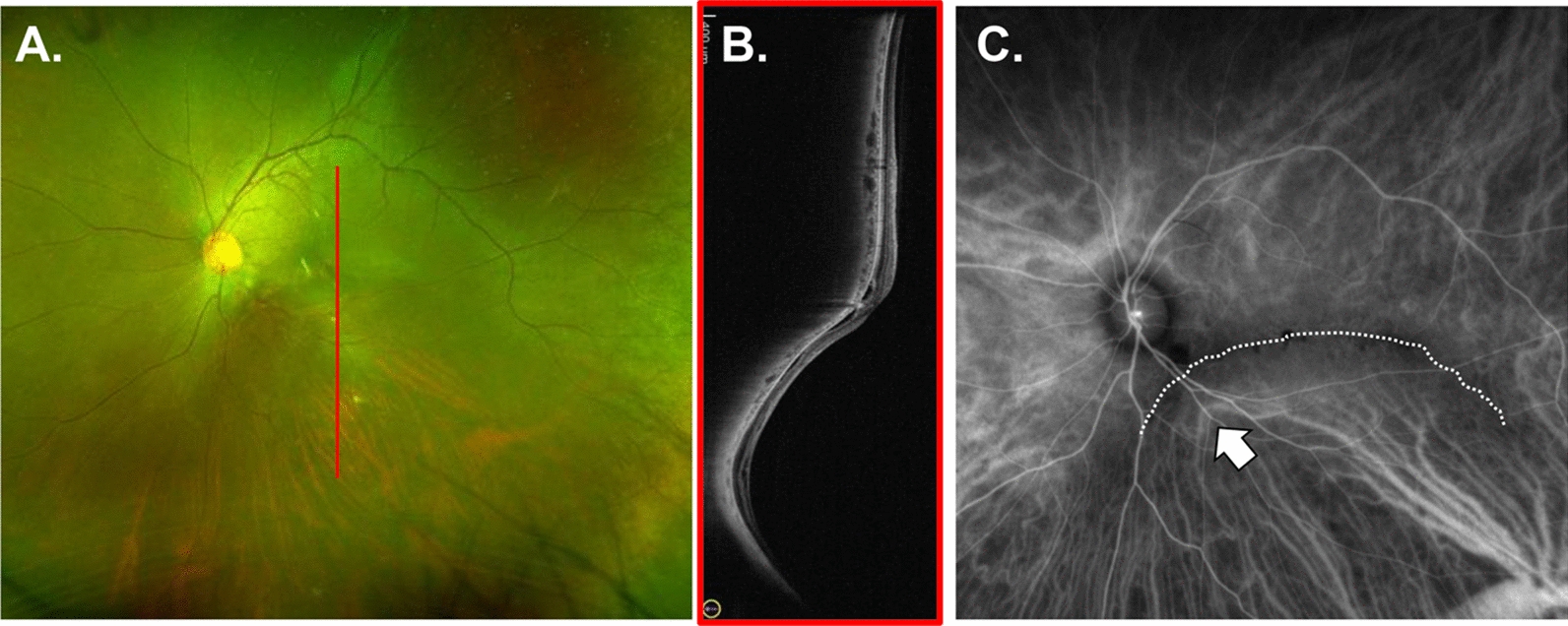
Haller’s vessels running patterns in a case of inferior posterior staphyloma. A 38-year-old male patient with serous macular retinal detachment secondary to IPS is presented (**A**: color SLO image; **B**: wide-angle OCT). Indocyanine green angiography shows Haller's vessels (white arrows) bending along the edge of the IPS (white dotted line) (**C**). IPS, inferior posterior staphyloma; OCT, optical coherence tomography; SLO, scanning laser ophthalmoscopy

Characteristic findings, such as an abnormal position of the vortex vein, an extraocular drainage channel of the choroid, and anastomosis of a single choroidal vessel through the posterior pole, have been observed in the healthy eyes [11] and those with high myopia [12]. In cases of the IPS, the choroidal large vessels may be flexed along the posterior staphyloma (Fig. 1). Therefore, the IPS may affect Haller’s vessel-running patterns (HVRPs).

In our previous report, we could not evaluate the macular shape in the areas distant from the fovea, such as the IPS, because of the limited imaging range of OCT [9]. Ultra-wide-field OCT (UWF-OCT), which has recently become available, has a wider imaging range and can be used to evaluate IPS [13–15].

Therefore, this study aimed to investigate the effect of the IPS on HVRP levels in healthy eyes using UWF-OCT.

## Methods

### Ethics statement

All the procedures conformed to the tenets of the Declaration of Helsinki and were approved by the Ethics Committee of Kagoshima University Hospital (approval number 180243, approval data: Dec. 18th. 2018). The requirement for informed consent was waived by the Ethics Committee of Kagoshima University Hospital owing to the retrospective nature of this study. An opportunity to opt out of the registry was provided to patients by disclosing the details of the study on the homepage of Kagoshima University Hospital.

### Participants

Records from healthy participants older than 20 years without known retinal diseases examined at the Ophthalmology Department of Kagoshima University Hospital between April 2021 and May 2022 were evaluated in a retrospective manner. First, every eye underwent a slit-lamp examination of the anterior segment and ocular fundus. We measured refractive error (RM8900, Topcon, Tokyo, Japan), decimal best-corrected visual acuity, and axial length (Tomey, Tokyo, Japan). Ultra-widefield fundus images were obtained using scanning laser ophthalmoscopy (Optos California, Optos PLC, Edinburgh, United Kingdom), en face OCT, and UWF-OCT (OCT-S1, Canon Corp, Tokyo, Japan). The best-corrected visual acuity was converted to the logarithm of the minimum angle of resolution of the visual acuity. The exclusion criteria were as follows: (1) eyes with blurred UWF-OCT images with signal strength lower than 4 (on a scale from 0 to 10); (2) eyes with a history of ocular surgery other than external ocular surgery, cataract surgery, or laser iridectomy; and (3) eyes diagnosed with uveitis, retinochoroidal disease, glaucoma, or malignant tumor.

### Evaluation of macular shape using UWF-OCT

**Fig. 2:**
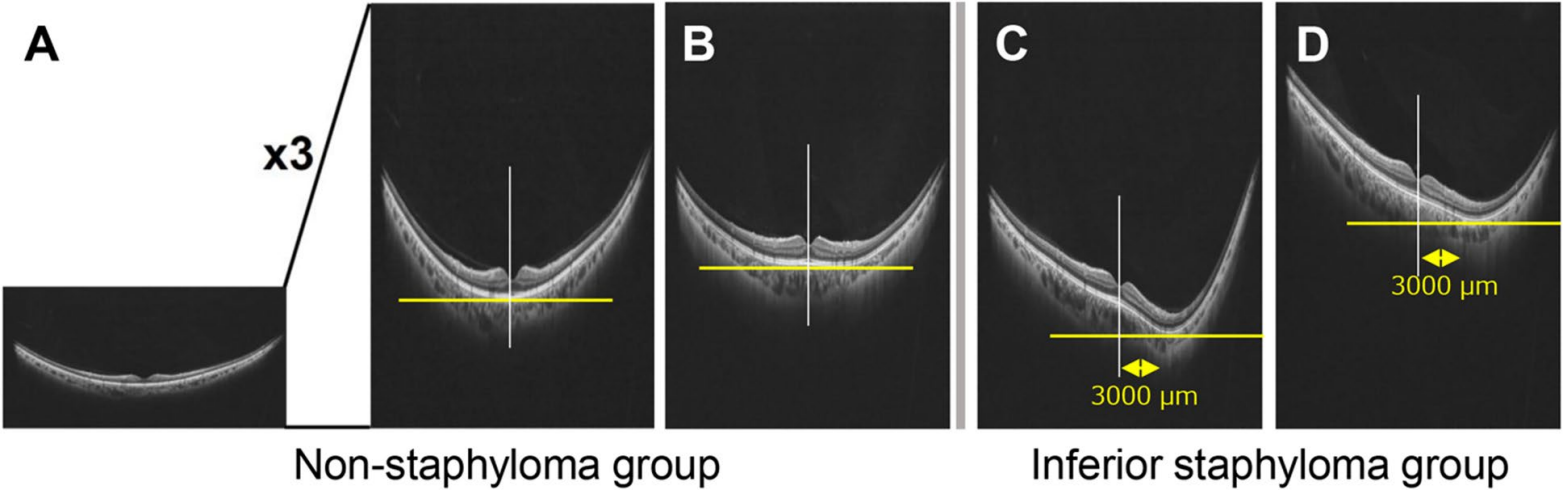
Definition of the non-inferior posterior staphyloma and inferior posterior staphyloma groups. The OCT images are magnified threefold vertically to enhance the macular shape (**A**). Two retina specialists (HT and RF) classify the patients into two groups: the non-IPS group, in which the inflection point of the macular shape coincides with the fovea (deviation within 3000 µm, **A**, **B**), and the IPS group, in which the position of the inflection point of the macular shape is deviated more than 3 mm below the fovea (**C**, **D**). In case of disagreement, a third retinal specialist (NM) makes a decision on line modification. IPS, inferior posterior staphyloma; OCT, optical coherence tomography

Similar to previous reports [9, 10], vertical images of radial UWF-OCT scans were included in the analysis. This is based on reports that vertical OCT images detect more IPS and dome-shaped macula than horizontal OCT images [5, 16]. The OCT images were magnified threefold vertically to enhance the macular shape (Fig. 2A). Two retina specialists (HT and RF) classified the patients into two groups: the non-IPS group, in which the inflection point of the macular shape coincided with the fovea (deviation within 3000 µm, Fig. 2A, B), and IPS group, in which the position of the inflection point of the macular shape deviated more than 3 mm below the fovea (Fig. 2C,D). We selected cases that corresponded to the IPS group by evaluating OCT images. The non-IPS group used as a control consisted of 113 healthy eye datasets used in previous reports [17].

### Propensity score matching (PSM)

Although factors correlated with macular choroidal thickness, such as sex, age, and ocular axis length, have been reported previously [18, 19], those that influence the running pattern of choroidal vessels are not well understood. Therefore, we matched participants based on age, sex, and ocular axial length, from a dataset of healthy participants [17].

To minimize the selection bias and control for confounding variables, we performed 1:1 PSM between the patient and control groups. Propensity scores were estimated using a logistic regression model with age, sex, and ocular axial length as covariates. Matching was performed using the nearest-neighbor method without replacement. We ensured that the matching caliper was set at 0.25 of the standard deviation of the logit of the propensity score to improve the quality of the matches.

### Evaluation of Haller’s vessels running patterns

HVRPs were evaluated using en face OCT images acquired in the OCT angiography mode of UWF-OCT, as previously described [20]. We defined Bruch’s membrane and the choroid–sclera interface as reference lines on en face OCT images. The OCT instrument's built-in software automatically created and manually modified these reference lines. We classified HVRP as the symmetry type, which did not cross the line between the fovea and optic disc. The upper vessels that crossed the line were of the upper-dominant type, and the lower vessels that crossed the line were of the lower-dominant type. Two retinal specialists (NM and RF) classified the HVRP as evaluators, and if the answers differed, a third retinal specialist (HT) made the final decision.

### Statistical analysis

The Mann–Whitney U test was used to compare the age, visual acuity, and ocular axial length between the non-IPS and IPS groups. Fisher's exact test was used to compare the sex and HVRP running patterns between the two groups. PSM was conducted using R version 4.2.0 (R Foundation for Statistical Computing, Vienna, Austria). Statistical significance was set at P < 0.05.

## Results

### Demographic information of participants

The demographic information of participants before PSM is shown in Table 1.

**Table 1:** Patient demographics before propensity score matching

	Inferior posterior staphyloma	Non-inferior posterior staphyloma	P-value*
Number of eyes	16	113	
Male:Female	8:7	53:60	0.648
Age (years)	30 (23–72)	53 (20–87)	< 0.001
Visual acuity (logMAR)	− 0.079 (− 0.18–0.15)	− 0.079 (− 1.08–0.52)	0.141
Axial length (mm)	25.37 (22.93–26.97)	23.68 (20.84–26.88)	< 0.001

*Fisher exact test is used for gender comparison between the two groups, and Mann–Whitney test is used to compare age, visual acuity, and axial length. The values for age, visual acuity, and axial length are shown as median (minimum–maximum)

A total of 16 eyes were classified as having inferior posterior staphyloma, whereas 113 eyes were categorized as non-inferior posterior staphyloma.

The ratio of male to female was 8:7 in the inferior posterior staphyloma group and 53:60 in the non-inferior posterior staphyloma group, showing no significant difference (*P* = 0.648). The age was significantly lower in the inferior posterior staphyloma group (median 30 years old, range 23–72) compared to the non-inferior posterior staphyloma group (median 53 years old, range 20–87) (*P* < 0.001).The logMAR visual acuity did not significantly differ between the two groups, with a median value of -0.079 (range -0.18–0.15) in the inferior posterior staphyloma group and -0.079 (range -1.08–0.52) in the non-inferior posterior staphyloma group (*P* = 0.141).The axial length was significantly longer in the inferior posterior staphyloma group (median 25.37 mm, range 22.93–26.97) compared to the non-inferior posterior staphyloma group (median 23.68 mm, range 20.84–26.88) (*P* < 0.001). The patient demographics expressed as mean ± standard deviation are provided in the Supplemental Table 1.

### Comparison of Haller’s vessels running patterns between groups before PSM

Among the 113 eyes in the non-IPS group, 65 (57.5%) had a symmetric pattern of Haller's vessels, 32 (28.3%) had an upper-dominant pattern, and 16 (14.1%) had a lower-dominant pattern.

In the IPS group, 14 eyes (87.5%) had an upper dominant pattern, and 2 (12.5%) had a symmetric pattern (Table 2).

**Table 2:** Choroidal vascular pattern in two groups before propensity score matching

	Upper dominant	Lower dominant	Symmetric	P-value
IPS	14	0	2	< 0.001
Non-IPS	32	16	65	

IPS: inferior posterior staphyloma

**Fig. 3:**
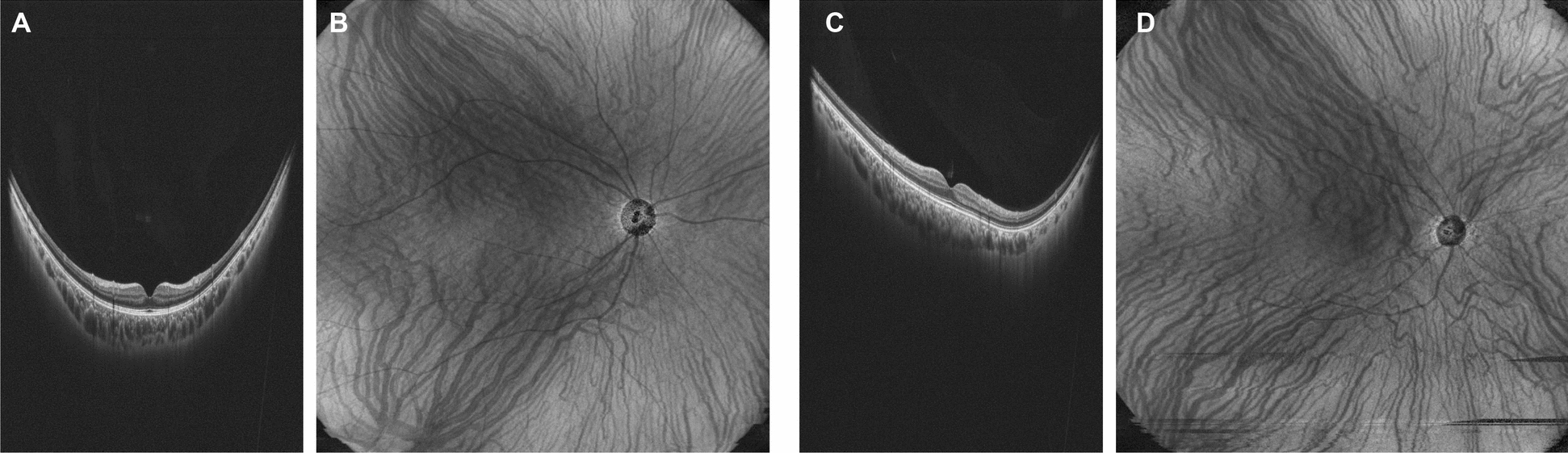
Representative cases from the non-IPS and IPS groups. A 24-year-old male in the non-IPS group. The axial length is 24.66 mm. The oculus is cone-shaped, centered on the fovea (**A**), and the Haller's vessel running patterns are symmetric (**B**). The IPS group includes one 25-year-old male. The axial length is 24.11 mm, and the inflection point is shifted inferior to the fovea (**C**). The Haller's vessel running pattern is an upper-dominant pattern (**D**). IPS, inferior posterior staphyloma

The two groups had a statistically significant difference in vascular running patterns (P < 0.001). Representative cases from the non-IPS and IPS groups are shown in Fig. 3.

### PSM

After the PSM, no significant differences were found in the sex, AL, and BCVA between the two groups (Table 3). Among the 12 eyes in the IPS group, 11 (91.7%) had an upper-dominant pattern symmetric pattern of Haller’s vessels and 1 (8.3%) had a symmetric pattern. Furthermore, in the 12 eyes of non-IPS group, 8 (66.7%) had a symmetric pattern of Haller's vessels, 2 (16.7%) had an upper-dominant pattern, and 2 (16.7%) had a lower-dominant pattern. Thus, even after PSM, the two groups had a statistically significant difference in vascular running patterns (P < 0.001, Table 4). The patient demographics after PSM expressed as mean ± standard deviation are provided in the Supplemental Table 2.

**Table 3:** Patient demographics after propensity score matching. The values for age and axial length are shown as median (minimum–maximum)

	Inferior posterior staphyloma	Non-inferior posterior staphyloma	P-value*
Number of eyes	12	12	
Male: Female	6:6	6:6	1.000
Age (years)	30 (23–72)	31 (21–66)	1.000
Axial length (mm)	24.5 (22.93–26.89)	24.72 (22.98–26.88)	0.885

**Table 4:** Choroidal vascular pattern in the two groups after propensity score matching

	Upper dominant	Lower dominant	Symmetric	P-value
IPS	11	0	1	< 0.001
Non-IPS	2	2	8	

IPS, inferior posterior staphyloma

## Discussion

In the present study, 87.5% and 91.7% of the HVRPs in the IPS group had an upper-dominant pattern before and after PSM, respectively. In the non-IPS group, both before and after PSM, the symmetric type was the most prevalent (57.5% before PSM and 66.7% after PSM), followed by the superior-dominant and lower-dominant patterns, respectively. Although there are differences in the analysis methods, these results are similar to those reported previously, in which 48.1–66.7% of normal eyes had a symmetric pattern of HVRPs, and the rest had an asymmetric pattern (upper-dominance was more common than lower-dominance) [21–24].

**Fig. 4:**
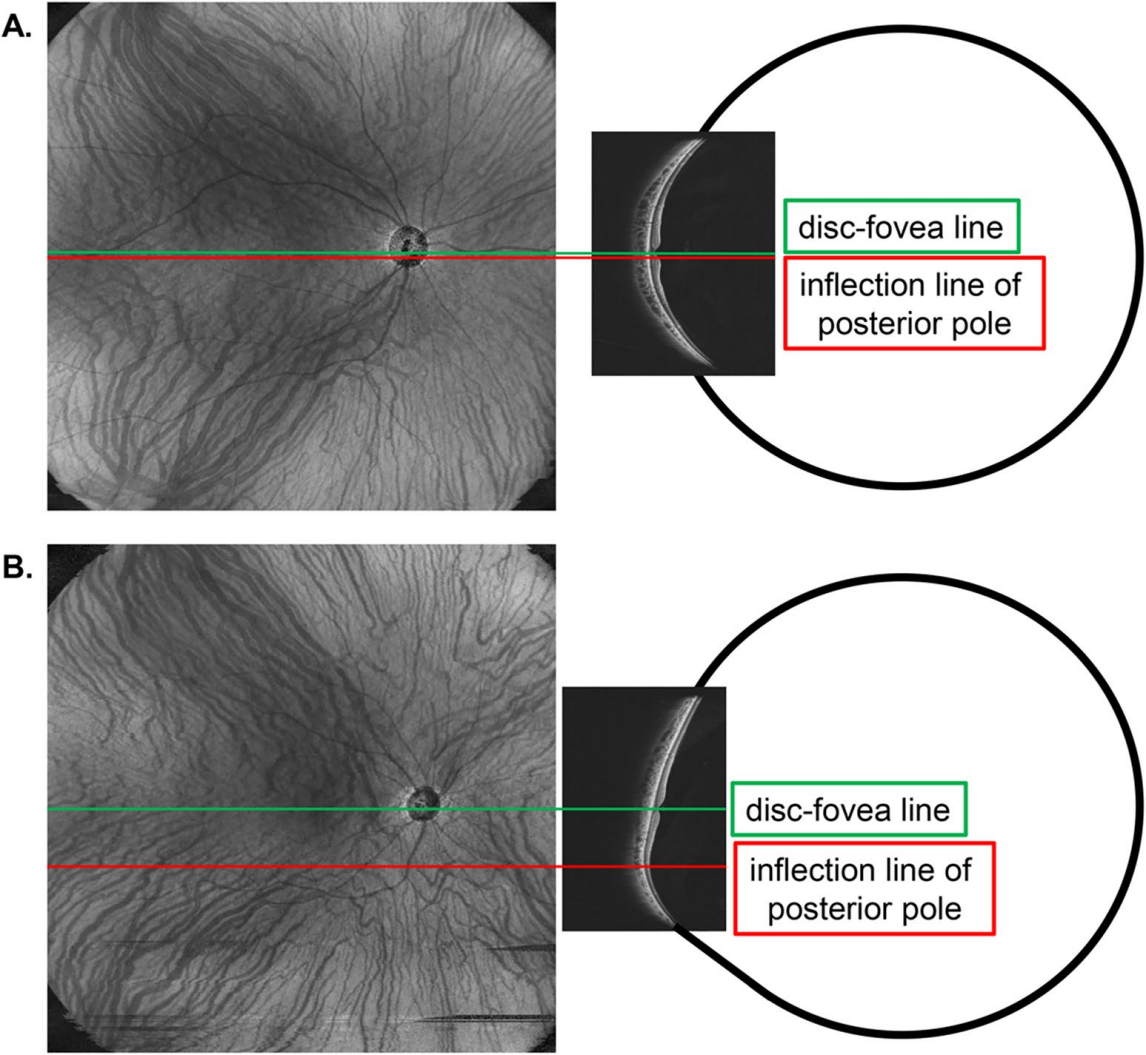
Haller’s vessels’ running pattern in an inferior posterior staphyloma. The reason for the predominance of an upper superior pattern in Haller's vessels running in IPS is that cases with a symmetrical running pattern before IPS occurring at a young age (**A**) may have their superior choroidal vessels stretched downward (**B**) as a result of the development of IPS as they grow older. In addition, in many cases of inferior posterior staphyloma, the inflection point of the posterior pole coincides with the location of the watershed zone of the choroidal vessels (**B**), suggesting that the presence of an inflection point may affect the flow of choroidal vessels. IPS, inferior posterior staphyloma

Considering this, we observed that most eyes with IPS had an upper-dominance of HVRPs, which differed from the results obtained in the non-IPS group. In addition, this trend persisted even after the PSM. The reason for this result may be the organization of the IPS. In the IPS, the lower part of the macula is deformed after a certain period when the eye is originally close to the regular sphere [25]. In this case, it is possible that the choroidal vessels, which have a symmetrical pattern that is common in normal eyes before the posterior pole shape changes (Fig. 4A), may have a more upper-dominant pattern in the IPS because the choroidal vessels above the macula are stretched downward by the IPS-like macular shape changes (Fig. 4B). An important question that remains unresolved is whether IPS induces changes in HVRP or if HVRP precedes and contributes to alterations in ocular shape. While our findings suggest a potential association between IPS and a superior-dominant HVRP pattern, the causal relationship remains unclear. Further longitudinal studies are necessary to determine whether vascular changes occur because of macular deformation in IPS or if pre-existing variations in HVRP contribute to the development of IPS. Understanding this temporal relationship would provide valuable insights into the pathophysiology of IPS and its vascular involvement.

As observed in ICGA findings showing increased vascular permeability [26], OCT findings in CSC [27], and certain types of AMD [28], the choroid plays a significant role in these pathological conditions. For example, choroidal thickness is increased in CSC and pachychoroid neovasculopathy [27] but reduced in retinal angiomatous proliferation [29]. Additionally, the ratio of choroidal luminal to stromal area is larger in CSC and PNV.

Regarding HVRP, previous reports have indicated that the proportion of HVRPs in CSC and AMD is often asymmetric and differs from that in normal eyes [22, 30, 31]. Recent studies have been increasingly clarifying these findings. Therefore, the eyes with IPS may be at a higher risk for CSC and AMD due to the highly asymmetric prevalence of HVRPs. The present results also showed that, in many cases of IPS, the inflection point of the posterior pole coincided with the position of the watershed zone of the choroidal vessels. This result is reasonable because the presence of blood flow across the inflection point makes it difficult for the blood to return hydrodynamically. This result also suggests that an inflection point may affect the HVRPs. In other words, when the posterior pole is flattened, it may be difficult for a watershed zone to form. Thus, such a shape may facilitate anastomosis, potentially increasing the risk of CSCs and other diseases. Future analyses of these eyes should be warranted (Supplementary Fig. 1).

This study had some limitations. First, it was difficult to identify cases of IPS in healthy subjects [9]. Therefore, a sufficient number of IPS cases could not be included in this single-center, retrospective study. In addition, factors associated with HVRPs are not fully understood. Thus, we created an age-, sex-, and axial length-matched non-IPS group because these factors are reportedly involved in macular choroidal thickness. Although there was a significant difference in the HVRPs between the IPS and non-IPS groups, further analysis in prospective studies and a larger number of cases is needed in the future. Next, the classification of HVRPs was performed subjectively, as in previous studies [20–24]. Since this approach has limitations in terms of reproducibility, it is necessary to reconsider HVRP evaluation using an objective assessment method in future studies. Finally, the data were obtained from a Japanese patient, who have been reported to have a high percentage of myopia [32–34]. Further studies involving other ethnic groups are required.

## Conclusions

In conclusion, the eyes with IPS are likely to have superior dominant HVRPs compared to the healthy eyes in non-IPS group. Macular shape may play a role in the HVRPs.

## Supplementary Information

Supplementary material 1.

Supplementary material 2.

Supplementary material 3.

## Data Availability

The datasets used and/or analysed during the current study are available from the corresponding author on reasonable request.

## Abbreviations

HVRP: Haller vessel running patterns
IPS: Inferior posterior staphyloma
OCT: Optical coherence tomography
