# Supplementary material for: Association between inferior posterior staphyloma on choroidal vessels running patterns in healthy eyes
Source: Int J Retina Vitreous. 2025 Mar 27;11:37. doi: 10.1186/s40942-025-00661-w (PMC11948877; doi:10.1186/s40942-025-00661-w)
Supplement: Supplementary file 1 — Supplementary material 1. [file 40942_2025_661_MOESM1_ESM.docx]

**Supplemental figure 1. Haller’s vessels’ running pattern in the eye with an flat macular shape**

**
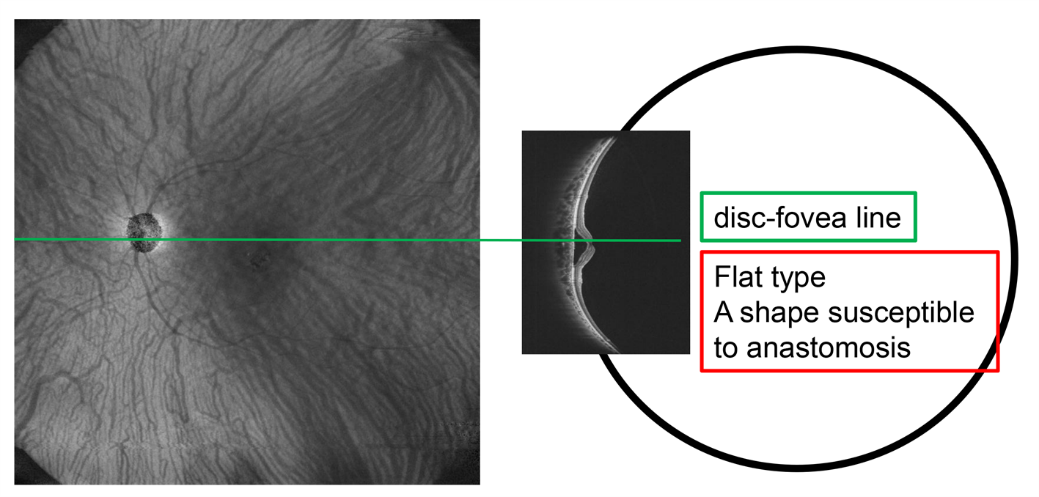
**

In many cases of inferior posterior staphyloma, the inflection point of the posterior pole coincides with the location of the watershed zone of the choroidal vessels, suggesting that the presence of an inflection point may affect the flow of choroidal vessels as in Figure 4. Conversely, we hypothesized that patients with a flat macular shape may be more susceptible to the choroidal vascular anastomosis and develop central serous chorioretinopathy because the inflection point is blunted.
