# Supplementary material for: Association between inferior posterior staphyloma on choroidal vessels running patterns in healthy eyes
Source: Int J Retina Vitreous. 2025 Mar 27;11:37. doi: 10.1186/s40942-025-00661-w (PMC11948877; doi:10.1186/s40942-025-00661-w)
Supplement: Supplementary file 2 — Supplementary material 2. [file 40942_2025_661_MOESM2_ESM.docx]

Supplemental Table1. Patient demographics before propensity score matching Baseline (Mean ± Standard Deviation, 95% Confidence Interval)

|  | **Inferior posterior staphyloma** | **Non-inferior**  **Posterior**  **staphyloma** | **p-value*** |
| --- | --- | --- | --- |
| **Number of eyes** | 16 | 113 |  |
| **Male:Female** | 8:7 | 53:60 | 0.648 |
| **Age (years)** | 32.2 ± 12.2  (25.7 – 38.7) | 52.8 ± 20.4  (49.2 – 56.7) | <0.001 |
| **Visual acuity**  **(logMAR)** | -0.07 ± 0.08  (-0.12 - -0.03) | -0.04 ± 0.10  (-0.076 - -0.024) | 0.141 |
| **Axial length** | 25.27 ± 1.27  (24.6 – 26.0) | 23.78 ± 1.22  (23.6 – 24.0) | <0.001 |

*: Fisher exact test was used for gender between the two groups, and Mann–Whitney test was used for age, visual acuity, and axial length.
