# Supplementary material for: Association between inferior posterior staphyloma on choroidal vessels running patterns in healthy eyes
Source: Int J Retina Vitreous. 2025 Mar 27;11:37. doi: 10.1186/s40942-025-00661-w (PMC11948877; doi:10.1186/s40942-025-00661-w)
Supplement: Supplementary file 3 — Supplementary material 3. [file 40942_2025_661_MOESM3_ESM.docx]

Supplemental table 2.

Patient demographics after propensity score matching (Mean ± Standard Deviation, 95% Confidence Interval)

|  | **Inferior posterior staphyloma** | **Non-inferior posterior**  **staphyloma** | **p-value*** |
| --- | --- | --- | --- |
| **Number of eyes** | 12 | 12 |  |
| **Male:Female** | 6:6 | 6:6 | 1.000 |
| **Age (years)** | 33.6 ± 13.8  (24.8 – 42.3) | 34.8 ± 15.5  (25.8 – 44.8) | 1.000 |
| **Axial length** | 24.83 ± 1.13  (24.1 – 25.5) | 24.88 ± 1.3  (24.1 – 25.7) | 0.885 |

*: Fisher exact test was used for gender between the two groups, and Mann–Whitney test was used for age and axial length.
